# Effects of Fly Ash and Hexadecyltrimethoxysilane on the Compressive Properties and Water Resistance of Magnesium Oxychloride Cement

**DOI:** 10.3390/polym15010172

**Published:** 2022-12-29

**Authors:** Bowen Guan, Zhenqing He, Fulu Wei, Faping Wang, Jincheng Yu

**Affiliations:** 1 School of Materials Science and Engineering, Chang’an University, Xi’an 710061, China; 2 China Construction Eighth Engineering Division Co., Ltd., Shanghai 200122, China; 3 Qinghai Transportation Holding Group Co., Ltd., Xining 810000, China

**Keywords:** fly ash, hexadecyltrimethoxysilane (HDTMS), magnesium oxychloride cement (MOC), compressive properties, water resistance

## Abstract

The application of magnesium oxychloride cement (MOC) is promising, but its poor water resistance seriously hinders its development and application. In this paper, we describe a new type of MOC with excellent water resistance, prepared using fly ash and hexadecyltrimethoxysilane (HDTMS). SEM, XRD, FTIR, TG/DSC, and other microscopic-scale studies were conducted to investigate the mechanism underlying the water-resistance enhancement of the new MOC. It was found that adding 20% fly ash and 3% HDTMS can strengthen the water resistance of MOC while retaining high mechanical properties. In particular, the residual coefficient remained at 0.91 after 7 days of immersion. This is because these two additives, when used together, can increase the content of the gelling 5-phase of MOC, as well as optimize the pore structure of MOC.

## 1. Introduction

MOC is an early-strength, fast-hardening, and air-hardening cement, which is prepared by mixing lightly burned magnesium oxide (MgO), magnesium chloride (MgCl_2_), and water (H_2_O) in a specific ratio. If the application of MOC can be promoted, its economic and ecological benefits could be effectively improved.

The hydration process of MOC is a condensation reaction of hydrated MgCl_2_ ions under alkaline conditions, which forms a 5-phase (5Mg(OH)_2_∙MgCl_2_∙8H_2_O) and a 3-phase (3Mg(OH)_2_∙MgCl_2_∙8H_2_O), determining the overall structure and strength of the material [1]. It has unparalleled advantages over other cementitious materials, including fast setting and hardening, very excellent bonding properties, good wear and corrosion resistance, good heat insulation, and flame retardancy, among other excellent characteristics. In addition, MOC also has good wrapping ability, and can maintain high mechanical strength when mixed with many other materials, such as fly ash, phosphate, metakaolin, and silica fume [2,3,4]. Despite this, its poor water resistance severely restricts the application range of MOC. It is generally believed that MOC is prone to strength decay in an aqueous environment, with the strength loss reaching about 70% when it is put into water for 28 days [5]. As a result, improving its water resistance is the most critical issue in the development and application of MOC.

Researchers have trialed various methods to enhance the water resistance of MOC, and the main directions are still promoting resistance against water intrusion and maintaining the invariability of the hydration products of MOC. Some researchers have started at the material level, improving the base ratio and curing temperature of MOC to enhance its density and, thus, its water resistance [6,7], while other researchers have enhanced the water resistance of MOC by curing with CO_2_ [8,9]. However, researchers are now more likely to achieve such effects by adding admixtures, such as fly ash [10], silica fume [11,12], phosphoric acid [13], limestone powder [14], polycarboxylic acid water-reducing agent, and corn starch [15,16,17].

During fuel combustion in thermal power plants, tiny ash particles are released as waste, called fly ash. It is well-known that burning a ton of coal can produce about 100–300 kg of fly ash [18]. China is a large user of coal and, if the large amount of fly ash produced is not treated, it can cause pollution of the environment. As fly ash can improve the performance of Portland cement, it has also been used in MOC modification research in recent years. Fly ash has been discovered to slow down the hydration process of MOC, lengthen its setting time, and increase its fluidity, but it has not been found to alter the creation of hydration products [10,19]. At the same time, a fly ash content of less than 30% can improve the water resistance of MOC, while also leading to a loss of strength [19]. Therefore, increasing the water resistance of materials while maintaining strength is a key direction of current research.

Due to the obvious disadvantages of inorganic additives and the fact that they are not easily available or produced in large quantities, research on organic additives has increased in recent years. The incorporation of organic additives can be divided into two categories. The first involves direct spraying or dipping of the finished cement into the liquid to form a film on the surface. However, a hydrophobic film alone is generally insufficient for promoting long-term water resistance of building engineering materials. As a result, many researchers have directly added organic additives to raw materials, in order to improve the water resistance of MOC [20,21]. Organosilanes—a class of water-repellent materials with excellent impregnation effect—have been studied by researchers as the focus of organic additives. Li et al. have prepared monolithic superhydrophobic MOC with better softening coefficients and compressive strengths by adding modified organosilanes directly during the preparation process [20]. Huang et al. have added hexadecyltrimethoxysilane (HDTMS) and P123 (poly(ethylene oxide)-poly(propylene oxide)-poly(ethylene oxide) and prepared hydrophobic MOC, using the amphiphilic property of P123 to weaken the effect of HDTMS on the MOC hydration reaction, thus increasing its resistance to water [21]. However, these methods also led to a loss of strength.

Although there have been several studies on the modification of MOC with fly ash and HDTMS, there have been no studies on improving its water resistance when they are both compounded. It has been reported that HDTMS has a modifying effect on fly ash, making it hydrophobic, thus providing a theoretical framework for this study. This paper aims to develop a formulation design of MOC with efficient water resistance performance, while conducting a comprehensive and in-depth study of the mechanisms involved in the impacts of fly ash and HDTMS on the performance of MOC.

## 2. Materials and Mix Design

### 2.1. Raw Materials

MOC is essentially composed of water, magnesium chloride hexahydrate (MgCl_2_∙6H_2_O), and magnesia powder that has been mildly burnt (MgO). Tianyi Refractory—where the raw material for MOC is produced—produces the slightly burned magnesia powder (purity, 90%) that, as determined by WB/T 1019–2002, the MgO has a 70% chemical reactivity [22]. Magnesium chloride (purity ≥ 98%) was selected for the magnesium chloride hexahydrate, which is produced by Damao Chemical Reagent Factory. Other additional materials were fly ash and hexadecyltrimethoxysilane (HDTMS). Table 1 and Table 2 detail the elemental compositions of the magnesium oxide, fly ash, and magnesium chloride, as determined with an X-ray fluorescence spectrometer (PW2400 WDXRF). The specific surface area of the MgO and FA powders were 300 m^2^·kg^−1^ and 330 m^2^·kg^−1^, respectively.

### 2.2. Mix Design

The control sample’s molar ratio was established with respect to our earlier research [23,24]. As the control sample, MOC paste was formed using a molar ratio of 8 MgO/MgCl_2_ and 13 H_2_O/MgCl_2_. Then, different doses of fly ash and HDTMS were added to the mixture in order to study the effects of the admixtures.

Two stages were involved in the preparation of the sample. First, fly ash (10%, 20%, 30%, or 40%) was mixed with the MOC slurry, and the optimal dosage of fly ash at the maximum compressive strength was taken as the standard, based on the compressive strength measured in the compressive test. Second, the ideal concentration of fly ash was combined with 0.1–3% of HDTMS (where the optimal amount of fly ash was obtained from the first stage of the experiment). The mixing ratios for MOC are given in Table 3. The mixing ratio of fly ash to HDTMS mainly referred to the previous literature [10,21,24].

## 3. Experimental Program

### 3.1. Preparation of Specimens

**Figure 1:**
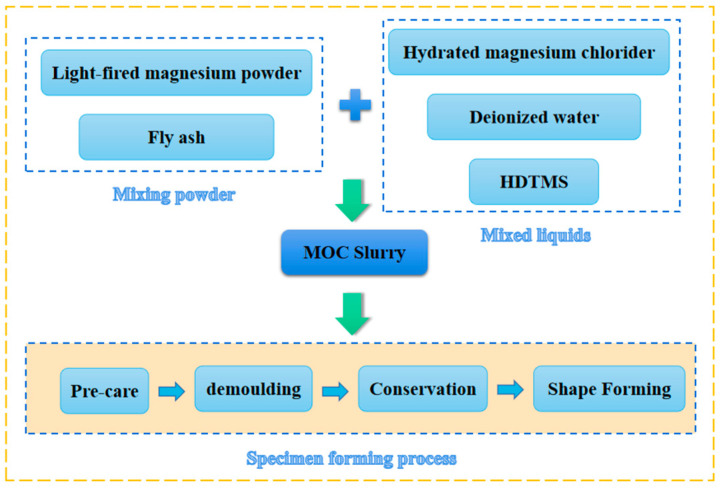
Flow chart for preparation of MOC specimens.

Before preparing the MOC specimens, a concentrated MgCl_2_ solution was prepared 24 h beforehand. First, MgO and fly ash were blended for 2 min in a cement paste mixer. Second, the MgCl_2_ solution was poured and the cement slurry mixer was started for mixing. Then, the fresh MOC paste was tested for fluidity and setting time. The paste was formed into a 40 mm × 40 mm × 40 mm cube mold to prepare the samples for mechanical testing. Then, a plastic cloth was placed over the cast MOC samples for 24 h at 25 °C during the initial curing process. The formed MOC samples were placed in an environment with 70% relative humidity for 7 days to cure, where the ambient temperature was controlled at about 25 °C. In contrast to the OPC curing process, acrylic curing does not require water spraying. The flow chart for preparation of MOC specimens is shown in Figure 1.

### 3.2. Methods of Testing

#### 3.2.1. Setting Time and Fluidity Tests

According to “Methods for testing uniformity of concrete admixture”, the fresh MOC paste was tested for fluidity [25]. Flow values for fresh MOC paste were determined by placing it in the standard cone and measuring its diameter at the base. The “Water Consumption, Setting Time and Stability Test Method for Standard Consistency of Cement” was used to measure the setting time [26]. Two samples were made for each group, using MOC paste and a conical ring.

#### 3.2.2. Compressive Strength and Water Resistance

After 7 days of air curing, MOC was tested for compressive strength. The loading speed of the compression test was 0.8 MPa/s, and the test results were the average of 6 groups.

The strength retention coefficient, *Wn*, derived using Equation (1), was calculated to assess the water resistance of MOC [23]:

(1) $$Wn=\frac{Scn}{Sc}$$

where *Scn* denotes the compressive strength of the samples (Immersion in water for 7 days), and *Sc* denotes the compressive strength of the samples (Air curing for 7 days).

After 7 days of air curing, well-cured MOC specimens were placed in water for 7 days at a temperature of 25 °C to determine *Scn*. Before performing the compression test, the samples were removed from the oven and allowed to stand and dry for 24 h at 40 °C.

#### 3.2.3. Microstructural Characterization

(1) X-ray diffraction (XRD)

After the strength test, the remaining sample was ground for microscopic testing. The XRD test angle was kept at 15–90°. The scanning speed was controlled at 0.5° per minute and the scanning time was 150 min. The samples to be observed were passed through a 75-mesh sieve. The X-ray diffractometer used was an AXS D8 ADVANCE.

(2) Fourier transform infrared spectroscopy (FTIR)

An FTIR spectrometer (BRUKER TENSOR II) was used to analyze the chemical composition of MOC composites between 4000 cm^−1^ and 400 cm^−1^. The samples to be observed were passed through a 200-mesh sieve.

(3) Thermal analyses

The MOC thermal degradation mechanism was derived through TG/DSC studies in the temperature range of 20–900 °C. This analysis allows for iodine excess analysis of components, by analyzing the change in mass with heating temperature. Before measurement, the sample was first ground into a fine powder and passed through a 20-mesh sieve, and a powder pattern of 20 mg passed through the sieve was taken for testing. The samples were heated, using a nitrogen flow, from 20 °C to 900 °C at a rate of 10 °C per minute during the tests. The synchronous TG/DTA thermal analyzer we used was produced by TA-Waters Company in the United States, and the specific model was Q600.

(4) Scanning electron microscopy test (SEM)

After 7 days of solidification, the sample fragments were coated with a conductive layer, and the microstructure was observed with a scanning electron microscope. The field emission scanning electron microscope we used was made by Hitachi, and the specific model was Hitachi S-4800.

(5) Pore size distribution analyses

In order to measure the pore size distribution, nitrogen adsorption was used. Samples were taken after 7 days of curing at 25 °C when the MOC had hydrated. Subsequently, the MOC specimen was broken to obtain smaller-volume specimens. Then, the samples were screened with a 0.6 cm square screen, in order to obtain tiny samples with particle size less than 0.6 cm. With anhydrous ethanol, we stopped the hydration reaction before baking the material at 60 °C for a period of 24 h. We removed the samples with tweezers, and then removed the impurities on their surface with a brush, ensuring that gentle movements were made when using the brush, in order to prevent damage to the surface of the sample. After that, we measured the pore structure using a physical adsorption instrument.

## 4. Results and Discussion

### 4.1. Effects of Fly Ash on MOC

#### 4.1.1. Setting Time and Fluidity

**Figure 2:**
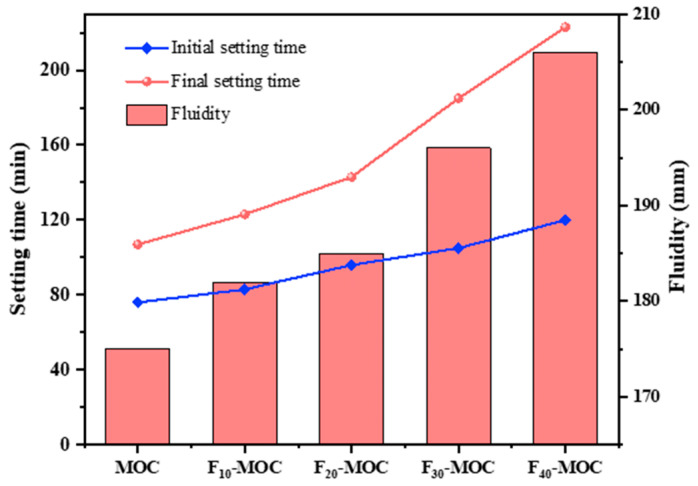
Fluidity and setting time of MOC mixed with fly ash.

As shown in Figure 2, fly ash affects the fluidity and setting time of MOC. Fly ash had a noticeable delaying effect on the setting time of MOC, which is consistent with the literature [10,19,27]. The initial setting time and final setting time of MOC reference samples were 76 min and 107 min, respectively, indicating that MOC has a fast setting rate. Compared to the setting time of MOC pure slurry without fly ash, the initial setting time of FA-MOC slurry was increased by 9.2–57.9%, and the final setting time was increased by 14.9–108.4%. Relevant studies have shown that the delay in setting time is related to the dilution effect of fly ash: as fly ash does not react with cement, the dilution effect after replacing cement slows down the overall reaction rate of MOC [13]. Moreover, Gurney has shown that fly ash hinders the establishment of bridges between hydration products, thereby delaying the condensation of MOC [28].

As shown in Figure 2, as the fly ash content increased, the fluidity of freshly mixed MOC changed from 175 mm to 206 mm, which increased by 3.9%, 5.5%, 11.5%, and 17.0%. This indicates that fly ash can enhance the machinability of MOC. The morphological effect of fly ash is one of the critical influencing factors. As the overall shape of fly ash is spherical, it can promote movement between adjacent particles [29]. Furthermore, the second reason is the dilution effect mentioned above, which slows down the flocculation of MOC, resulting in better fluidity of fresh MOC. Thus, fly ash can successfully increase the fluidity of MOC slurry.

#### 4.1.2. Compressive Strength

**Figure 3:**
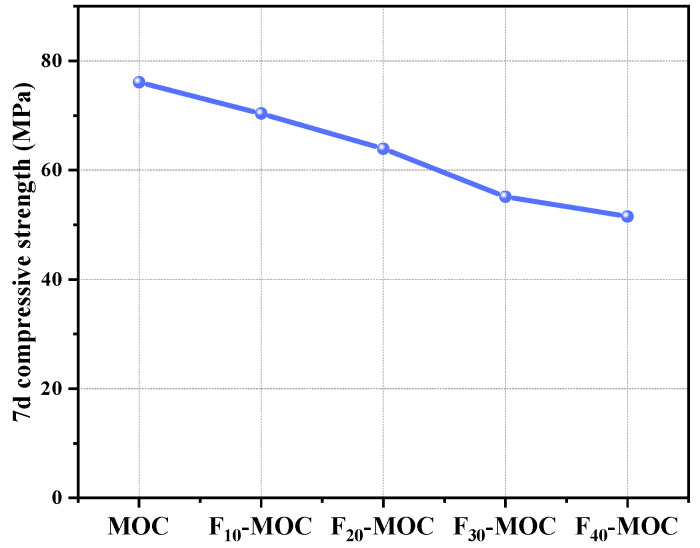
Compressive strength of fly-ash–MOC composite binary system.

Figure 3 shows the 7-day compressive strength of MOC mixed with different proportions of fly ash. As the fly ash content increased, the 7-day compressive strength of MOC specimens decreased, and the MOC samples mixed with 10–40% fly ash were 7.55%, 16.0%, 27.6%, and 32.3% lower than the reference samples. This is mainly due to the fly ash partially replacing magnesia, thus diluting the reaction system, breaking the bridge between the hydration products, weakening the system structure and, finally, resulting in a loss of mechanical characteristics [24,30,31,32].

#### 4.1.3. Water Resistance

**Figure 4:**
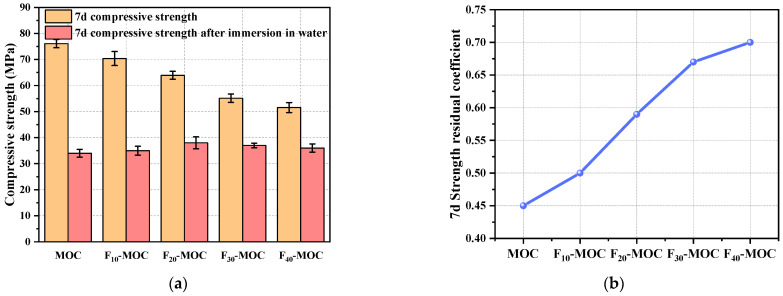
Water resistance of fly-ash–MOC composite binary system: (**a**) 7 d compressive strength; and (**b**) 7 d strength residual coefficient after immersion in water.

The water resistance of MOC can be characterized using the strength residual coefficient. The strength and strength residual coefficient of MOC with different fly ash contents after curing for 7 days and soaking for 7 days are shown in Figure 4.

As shown in Figure 4a, the compressive strength of MOC after immersion was lower than before water immersion. This is mainly because the 5-phase in MOC is hydrolyzed in water to generate Mg(OH)_2_ with lower strength, thus reducing its structural strength [33].

From Figure 4b, with the increase in fly ash content, the strength residual coefficient of MOC showed an increasing trend. For the MOC with fly ash contents of 10%, 20%, 30%, and 40%, the strength residual coefficient was higher than that of the reference sample after immersion (increased by 11.1%, 31.1%, 48.9%, and 55.6%, respectively), demonstrating that fly ash can improve the water resistance of MOC.

**Figure 5:**
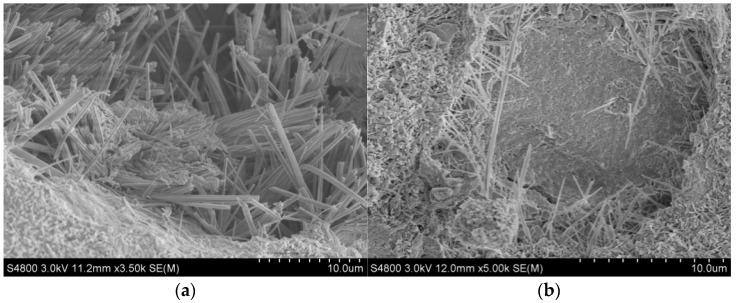
SEM images of MOC benchmark sample and 20% fly-ash-doped MOC sample after immersion in water: (**a**) MOC benchmark; and (**b**) MOC sample mixed with 20% fly ash.

Scanning electron microscopy was performed on the reference sample of MOC and the MOC specimen mixed with 20% fly ash after soaking (Figure 5). Comparing Figure 5a,b, the reference sample mainly contained needle-like 5-phase, which were separated from each other, resulting in the formation of a spatial network structure with more pores in the entire reference sample. Such a structure cannot prevent the intrusion of water and so the MOC sample will be greatly affected by water (especially its mechanical properties), where the coefficient is only 0.45. From Figure 5b, compared with the reference sample, we found that the MOC specimens mixed with 20% fly ash formed more gelatinous 5-phase and the interior was relatively dense, consistent with the literature [24]. This structure can effectively prevent the intrusion of water, such that the MOC has good water resistance, and its residual strength coefficient was increased to 0.59. When fly ash is added to MOC under alkaline conditions, it produces amorphous aluminosilicate gel, which improves the water resistance of the material. This is due to the pozzolanic reaction between SiO_2_ and Al_2_O_3_ in fly ash, which produces amorphous aluminosilicate gel. As a result of their large surface area, the well-dispersed fly ash particles are likely to embed themselves in the microstructure of the MOC crystals with the aluminosilicate gel, forming a microstructure similar to the microstructure of the MOC crystals.

To some extent, the 5-phase is protected by the surrounding fly ash and the aluminosilicate gel network. In addition, some studies have shown that the growth of Mg(OH)_2_ in the MOC slurry can be inhibited, to a certain extent, by incorporating fly ash, which means that fly ash can inhibit the hydrolysis of the 5-phase. Therefore, the enhanced water resistance of MOC slurry can also be attributed to the inhibition of the growth of Mg(OH)_2_ by fly ash [19].

### 4.2. Effects of Fly Ash and HDTMS on MOC

#### 4.2.1. Setting Time and Fluidity

**Figure 6:**
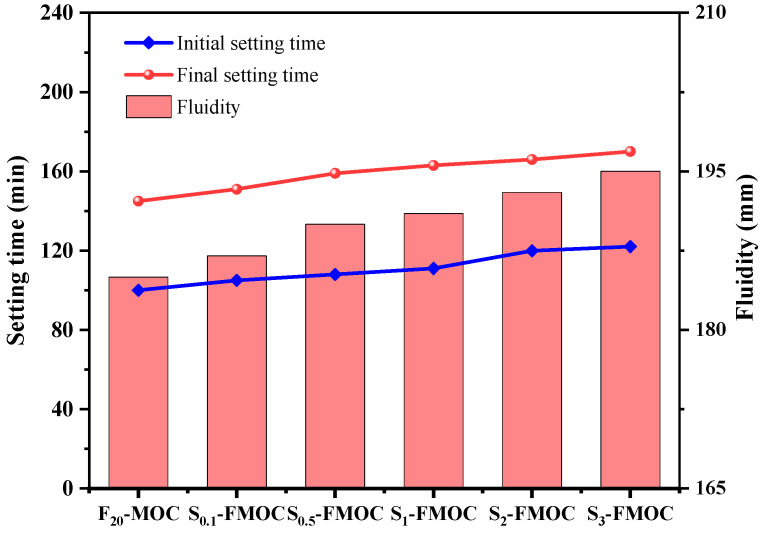
Setting time and fluidity of compound material–magnesium oxychloride cement composite ternary system.

**Figure 7:**
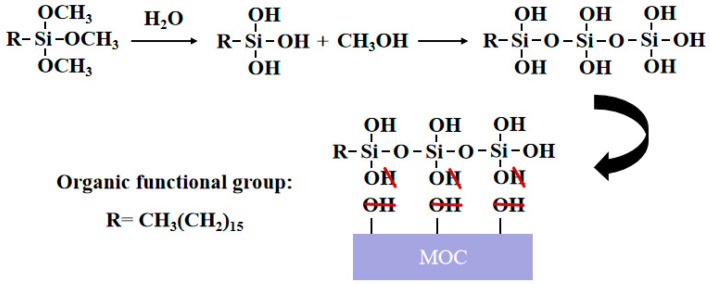
Hydrolysis process of HDTMS [18].

**Figure 8:**
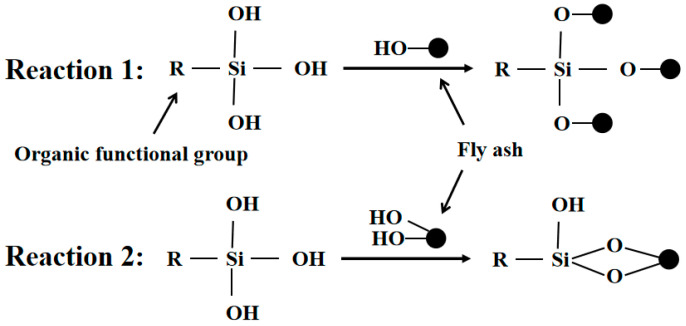
Reaction process between HDTMS and fly ash.

Figure 6 displays the fluidity and setting time of the newly modified MOC. The addition of 20% fly ash and different proportions of HDTMS affected the setting time of the freshly mixed MOC, and the amount of HDTMS content increased along with the initial and final setting time. When the HDTMS content increased from 0% to 3%, the initial setting time was prolonged by 5.1%, 8.3%, 11.2%, 20.5%, and 22.3%, and the final setting time was prolonged by 4.1%, 9.7%, 12.4%, 14.5%, and 17.2%, respectively. This indicates that the rate of the hydration reaction of MOC was reduced, mainly as HDTMS will hydrolyze itself when contacting water (as shown in Figure 7). The polysiloxane formed by hydrolysis will coat the surface of MgO to prevent water-soluble intrusion, which slows down the hydration reaction between MgCl_2_ solution and MgO. The initial and final setting times are increased as a result. Second, HDTMS modifies the fly ash; the specific mechanism is shown in Figure 8. The silanols hydrolyzed by HDTMS and the hydroxyl groups on the surface of the modified fly ash undergo condensation and dehydration reactions. The silane coupling agent will be connected to the fly ash surface by a chemical bond to form a coupling agent monomolecular layer, such that the fly ash will transition from hydrophilic to lipophilic [34]. The dispersion effect will become more obvious, such that bridges cannot be established between cement particles and, so, the setting time will be prolonged. Similarly, the unmodified fly ash also prolongs the setting time of MOC.

It can be seen from Figure 7 that with the increase in the content of HDTMS, the fluidity of the fresh MOC will also increase. Theoretically, HDTMS is added to the MOC system such that a greater number of methyl groups will be present, resulting in a more hydrophobic MOC system.

During the hydrolysis process of HDTMS, methoxy groups are replaced by hydroxyl groups (as shown in Figure 3), following which the hydroxyl groups interact with each other to form a polysiloxane chain structure. In the same way, the hydroxyl group of HDTMS will react with the hydroxyl group of MOC and undergo dehydration condensation. The generated non-polar –Si–R groups can repel each other with hydrogen atoms in water, which causes the MOC to become hydrophobic after internal mixing with silane, reducing the bonding property inside the fresh MOC paste [21]. HDTMS also leads to modification of the fly ash from hydrophilic to lipophilic, and the spherical character of fly ash facilitates the flow of MOC slurry, such that the fluidity will be overall enhanced and the paving circle diameter will be increased.

#### 4.2.2. Compressive Strength and Strength Residual Coefficient Test

**Figure 9:**
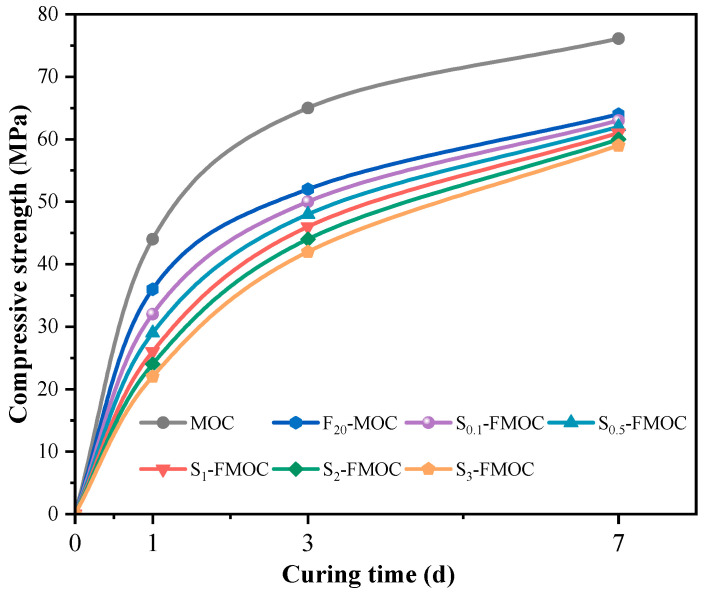
Effects of fly ash and HDTMS on the development of compressive strength of MOC.

Figure 9 shows the development of compressive strength of MOC-modified samples under different curing ages. The development trend of the compressive strength of the MOC under the mixed condition was basically similar to that of the MOC reference sample, indicating that the mixture of HDTMS and fly ash did not change the hydration process of the MOC. It is also worth noting that the compressive strength of the compound-modified MOC was lower than that of the MOC benchmark at any age. Adding fly ash and HTDMS will cause the MOC to respond more slowly, resulting in reduced compressive strength for MOC at the same primary ratio and age. The fly ash and HDTMS may not promote the hydration of MOC in air curing conditions for 7 d and reduce the content of cementitious materials. The figure shows that, after 7 days, both the modified MOC and the standard MOC experience a period of strength increase, but this increasing trend will eventually flatten out.

**Figure 10:**
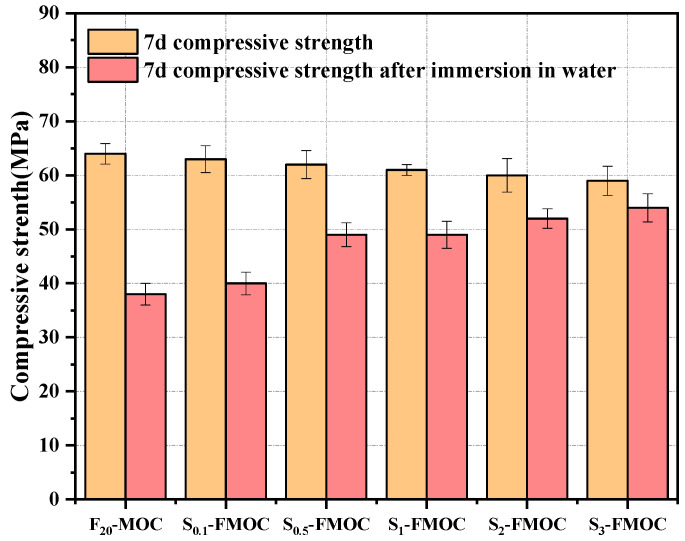
7 d compressive strength of compound material–magnesium oxychloride cement composite ternary system.

The compressive strengths of the MOC combined with fly ash and HDTMS after 7 days of curing and after 7 days submerged in water are shown in Figure 10. Compared with FA-MOC, the compressive strength of MOC-modified samples decreased with the increase in HDTMS content. This is due to HDTMS being easy to hydrolyze and condense to form polysiloxane, forming many hydrophobic substances. These hydrophobic substances coat the surface of MOC to prevent further contact between thecement and the aqueous solution, thereby affecting the compressive strength of MOC [35]. The HTDMS-modified fly ash was also dispersed in the MOC simultaneously. Compared with unmodified fly ash, it can be more uniformly dispersed in MOC, blocking the connection between hydration products and weakening the strength.

**Figure 11:**
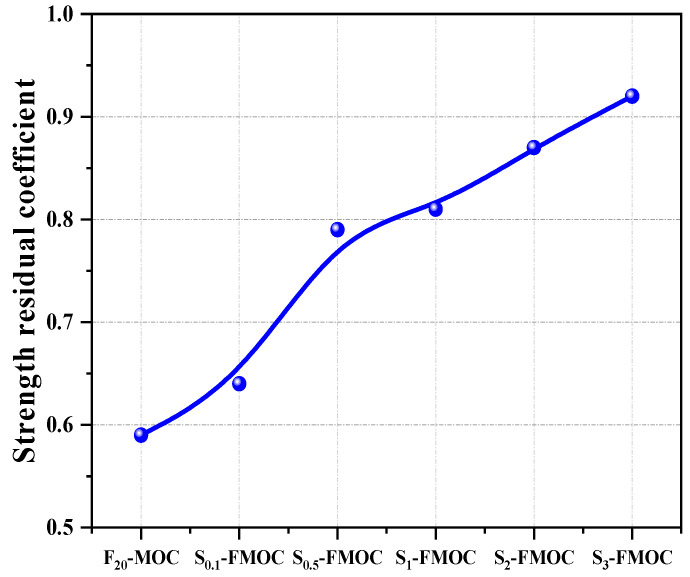
Strength residual coefficient of composite material–magnesium oxychloride cement composite ternary system after water immersion.

It can be seen from Figure 11 that with the increase in HDTMS content, the combined effect of fly ash and different ratios of HDTMS increased the residual strength coefficient of MOC. When 20% fly ash was added, the residual strength coefficient of MOC after soaking was increased to 0.59. After adding 0.5%, 1%, 2%, and 3% of HDTMS, the residual strength coefficients increased to 0.79, 0.81, 0.87, and 0.92, respectively, which were 33.9%, 37.3%, 47.5%, and 55.9% higher than that of F_20_-MOC. Comparing Figure 10 and Figure 11, it is not difficult to see that FA-MOC incorporated with HDTMS presented excellent comprehensive mechanical properties and water resistance at dosages of 1%, 2%, and 3%. These results show that HDTMS can effectively prevent the decomposition of the 5-phase in an aqueous environment. As MOC has a higher residual strength coefficient after soaking in water, it is more advantageous for long-term applications.

#### 4.2.3. X-ray Diffraction (XRD)

**Figure 12:**
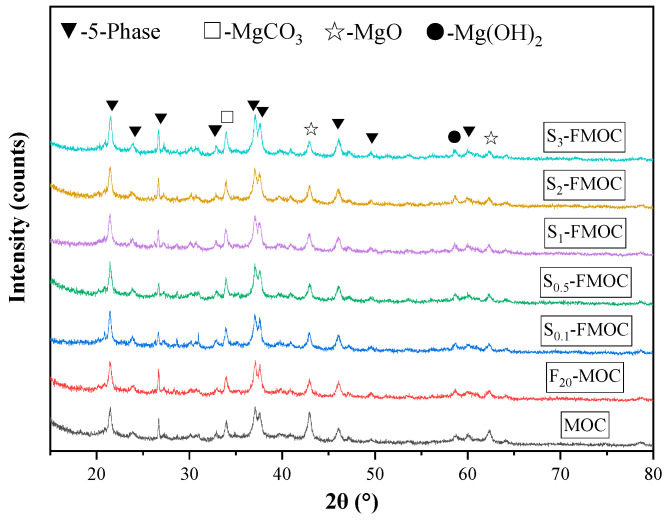
XRD patterns of hardened multi-doped MOC.

The XRD pattern of the MOC sample is shown in Figure 12. The findings demonstrate that fly ash and HDTMS increased the fraction of 5-phase in the hardened MOC to varying degrees, while simultaneously reducing the amount of unreacted magnesium oxide present in the combination.

As shown in Figure 12, the composition of the hydration products was unaffected by the addition of fly ash and HDTMS to the MOC slurry. The primary hydration products were 5-phase and MgCO_3_, with diffraction at 2θ = 43° and 63°. The peaks indicated the presence of unreacted magnesium oxide. After adding 20% fly ash to MOC, the content of the 5-phase was relatively increased. The increase in hydration products indirectly proves that fly ash can make the hydration process more complete. Fly ash is added to the MOC system, filling the pores of the pastes. This is because the average particle size of fly ash is lower than that of magnesia particles. Additionally, the fly ash between the particles of magnesium oxide can separate the aggregated particles of magnesium oxide [24]. Consequently, additional hydration products are produced as the water enclosed in the magnesium oxide particles is freed and takes part in the hydration process. From Figure 12, it can be seen that the incorporation of HDTMS into FMOC led to a significant increase in the content of 5-phase, due to the polysiloxane formed by the hydrolysis of HDTMS, the coupling product of silanol and magnesium oxide formed by the hydrolysis of HDTMS, and the modified fly ash. Both have a hydrophobic dispersion effect, divide the hydration area, make the hydration more thorough, and thus produce more hydration products. A small amount of magnesium carbonate was found in the XRD pattern, due to carbon dioxide entering the interior of the MOC sample along the pores and reacting with Mg(OH)_2_ to form MgCO_3_.

#### 4.2.4. FTIR Analysis

**Figure 13:**
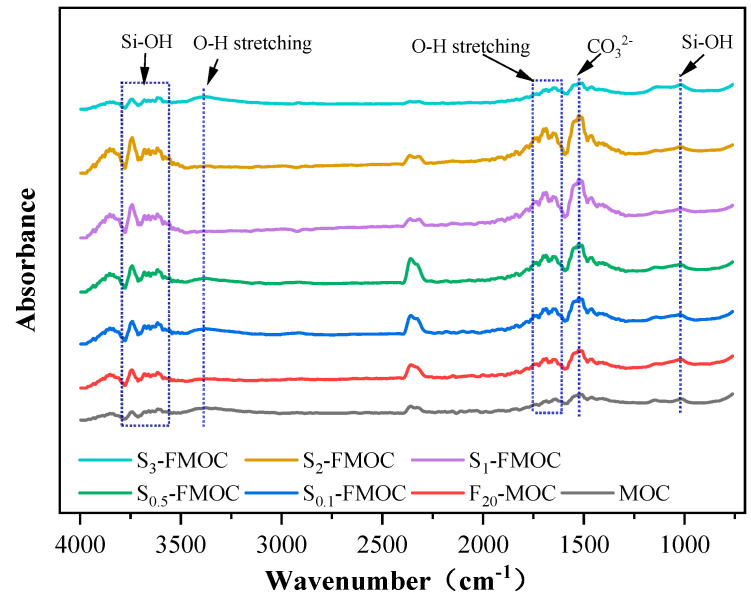
Infrared spectra of hardened and multi-doped MOC.

The infrared spectra of fly ash, HDTMS, and MOC ternary systems at different ratios are shown in Figure 13. Seven samples (MOC, F_20_-MOC, S_0.1_-FMOC, S_0.5_-FMOC, S_1_-FMOC, S_2_-FMOC, and S_3_-FMOC) allowed for the detection of functional groups. As shown in the figure, more intense peaks appeared at 3742 cm^−1^, 3679 cm^−1^, 3657 cm^−1^, 3639 cm^−1^, and 3613 cm^−1^, indicating that the O–H bond of crystal 5 phase and Mg–Cl–Si–H present stretching vibrations [36].

The ratio of the 5-phase was substantially higher when 20% fly ash was added into the MOC, which was essentially consistent with the earlier XRD investigation. The addition of fly ash promotes the hydration reaction of magnesium oxychloride cement, thus increasing the 5-phase. The content of CO_3_^2-^ also increased (with a strong peak at the wave number 1523 cm^−1^), derived from MgCO_3_. During the hydration reaction of MOC, Mg(OH)_2_ is generated as a by-product. Furthermore, the 5-phase encountering water in the MOC will decompose to form Mg(OH)_2_ during the curing process. When preparing the magnesium oxychloride cement slurry, a small amount of CO_2_ will be brought into the slurry, and this Mg(OH)_2_ will meet with CO_2_ to form MgCO_3_.

HDTMS was added to FMOC at concentrations of 0–2%. With its increasing concentration, the ratio of the 5-phase gradually increased, as well as the amount of amorphous Mg–Cl–Si–H gel. The results demonstrate that the combined action of HDTMS and fly ash makes the hydration reaction of MOC more complete, as well as increasing the proportion of amorphous 5-phase, consistent with the conclusion obtained using XRD analysis. This is because the polysiloxane formed by the hydrolysis of HDTMS wraps and divides the hydration reaction area, thus making the hydration reaction more thorough. As can be seen from Figure 13, the content of CO_3_^2-^ also increased with an increase in HDTMS content, as the 5-phase and Mg(OH)_2_ are in a dynamic equilibrium of mutual transformation, and the incorporation of HDTMS makes the hydration reaction of magnesia cement more thorough. It increases the proportion of the 5-phase and, so, the content of Mg(OH)_2_ in dynamic equilibrium with the 5-phase will also increase. At the same time, the hydration reaction can also produce Mg(OH)_2_, such that the degree of progress of the hydration reaction will also directly affect the Mg(OH)_2_ content. An interesting phenomenon is that the content of 5-phase, CO_3_^2-^, and Mg–Cl–Si–H gel phase was significantly reduced in MOC mixed with 3% HDTMS and 20% fly ash. There are two possible explanations for the phenomenon. The first is due to experimental errors or errors caused by human beings. Second, with a large amount of HDTMS added, the magnesium oxide particles are entirely wrapped in the polysiloxane produced by the hydrolysis of HDTMS. This affects the formation of hydration products and reduces the 5-phase, CO_3_^2-^, and Mg–Cl–Si–H gel phase contents.

From Figure 13, it can be seen that there was a large peak at 2400–2000 cm^−1^. Verification revealed that this peak corresponds to carbon dioxide, which the FTIR instrument is responsible for producing. By reducing the time between background acquisitions, this issue may be resolved [23].

#### 4.2.5. TG and DSC Analysis

**Figure 14:**
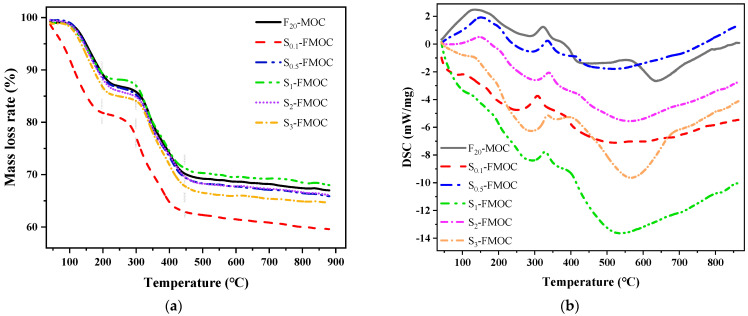
TG/DSC curves of hardened and multi-doped MOC, (**a**) TG curves, (**b**) DSC curves.

TG and DSC were employed in order to analyze the hydration products of MOC in greater detail, as shown in Figure 14.

During the analysis, the weight loss and differential calorific value of MOC samples were measured. As shown in Figure 14a,b, when fly ash was mixed with HDTMS simultaneously, the weight loss process of MOC was not affected, but the differential heat will change, to a certain extent. In Figure 14a, the TG curves of MOC samples doped with fly ash and different proportions of HDTMS are shown. The mass loss ratio was the same for all MOC samples (including modified and reference samples) in the 200–500 °C range, indicating that the combined action of fly ash and HDTMS did not affect the mass loss process of MOC. Looking at Figure 14b again, it can be seen that, at 200–500 °C, the exothermic peak of the compound-modified MOC sample was gentler than that of the MOC reference sample. This phenomenon implies that many Mg–Cl–Si–H gels were formed in the modified MOC specimens that were compounded with fly ash and HDTMS. This also demonstrates that, when fly ash and HDTMS are used together in a compound, the proportion and form of the hydration products (crystalline or amorphous) can significantly differ, causing the shape and intensity of the DSC peaks to change.

#### 4.2.6. SEM Observation

**Figure 15:**
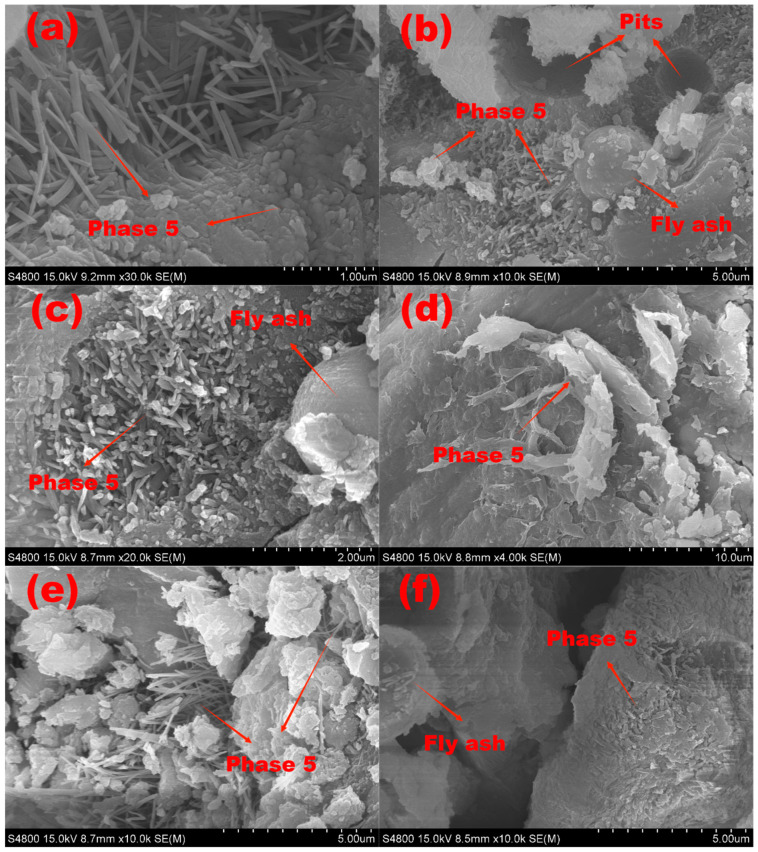

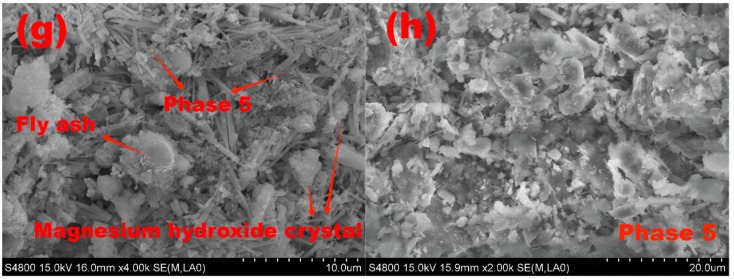
SEM images of modified MOC. Not soaked in water: MOC reference sample (**a**); F_20_-MOC (**b**); S_2_-FMOC (**c**–**e**); and S_3_-FMOC (**f**). Water immersion: S_2_-FMOC (**g**,**h**).

Through scanning electron microscopy, the morphologies of the selected samples were examined. Typical SEM images are displayed in Figure 15, from the MOC benchmark sample (a), F_20_-MOC (b), S_2_-FMOC (c, d, e), S_3_-FMOC (f) cured in isolated air for 7 days, and S_2_-FMOC (g, h) after 7 d isolation air curing and 7 d water immersion treatment.

Numerous whiskers or crystals resembling needles can be seen in Figure 15a, around which some bulk crystals were produced. The 5-phase developed in the MOC reference sample was in a needle-like crystalline phase. These 5-phase crystals form along the interior surfaces of the macropores in the hardened MOC samples, enhancing the MOC’s overall mechanical characteristics, due to its microstructure. From Figure 15b, the spherical shape observed in the SEM image is the fly ash particle that did not participate in the hydration reaction of MOC. The two surrounding spherical pits are also caused by fly ash particle detachment. The unreacted fly ash particles are distributed throughout the MOC system, dividing the reaction system of the fresh MOC and delaying the coagulation rate. Figure 15b further shows some fly ash particles that have not yet reacted with 5-phase growth on their surface.

Figure 15c–e show scanning electron microscope images of S_2_-FMOC. It can be seen from these images that when the fly ash and HDTMS were mixed, 5-phase can be obtained in the form of needle sticks, flakes, and gel. Additionally, only a few whiskers of 5-phase show up on the amorphous phase surface, resulting in less network support structure in the MOC. As a result, the MOC’s strength is reduced. However, the Mg–Cl–Si–H gel may successfully minimize the microcracks in the MOC sample. Meanwhile, the 5-phase coexists with the Mg–Cl–Si–H gel, thus shielding the MOC’s interior components from water assault and increasing water resistance.

Figure 15g,h are the SEM images of S_2_-FMOC after water immersion. Compared with Figure 15c–e, the internal microscopic morphology of S_2_-FMOC after water immersion had changed. After immersion in water for 7 days, the 5-phase of the reference sample partly became loose scaly Mg(OH)_2_, as part of the clustered 5-phase. Some remaining 5-phase crystals can be clearly seen. Comparing with Figure 5b, it can be seen that the modified MOC after water immersion retained a large proportion of the 5-phase, indicating that the combined action of fly ash and HDTMS can effectively delay the decomposition of the 5-phase, such that a part of the 5-phase is retained. Therefore, the water resistance is enhanced.

Theoretically, the 5-phase will gradually evolve into loose Mg(OH)_2_ in an aqueous environment, which is highly detrimental to the water resistance of MOC. However, the chemical and physical properties of fly ash and HDTMS allow most of the 5-phase to be retained, and the fly ash is uniformly dispersed in the MOC hardened slurry, blocking part of the connected pores of the MOC system and preventing moisture intrusion. The complexes formed by the hydrolysis of HDTMS cover the surface of the 5-phase and other hydration products to form a hydrophobic film. The hydrolyzed products of HDTMS chemically react with the MOC, thereby firmly fixing the hydrophobic film on the surface of the MOC. In addition, HDTMS modifies part of the fly ash to make it hydrophobic, where the modified fly ash on the MOC surface has a better water-blocking function.

#### 4.2.7. Pore Structure Analysis

**Figure 16:**
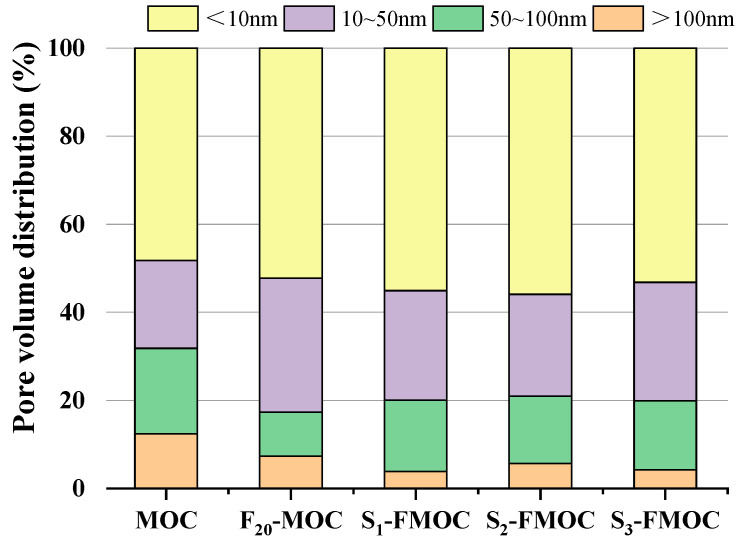
Pore volume distribution of hardened multi-doped MOC.

In order to determine the pore structure of MOC, nitrogen adsorption was used to measure the pore size. Figure 16 depicts the distribution of pores in the MOC at various doping ratios.

By analyzing the percentage of pore-size volume distribution, it was found that small pore sizes increased when fly ash was mixed into the MOC, and the proportion of harmful pores decreased. This is due to fly ash being uniformly dispersed in the MOC slurry, which divides a large number of large-diameter pores and increases the proportion of gel pores. This reduction in harmful pores enhances the water resistance of MOC.

Figure 16 details the decrease in the proportion of high pore sizes in the MOC sample following the addition of fly ash and HDTMS, proving that the pore structure has, once again, been optimized. At the same time, it can also be seen that the water resistance of the modified MOC has a specific relationship with the pore structure. The more significant the proportion of gel pores occupied, the better the water resistance of the MOC. The smaller pore size (gel pore) combined with the hydrophobicity of various reaction products after the hydrolysis of HDTMS acts together, making it difficult for water to penetrate the MOC, effectively improving the water resistance. Meanwhile, the content of gel micropores (<10 nm) is related to the 5-phase and the gel pores in the Mg–Cl–Si–H gel, which also indirectly indicates that the content of Mg–Cl–Si–H gel in the hydration product will increase after adding fly ash and HDTMS.

**Figure 17:**
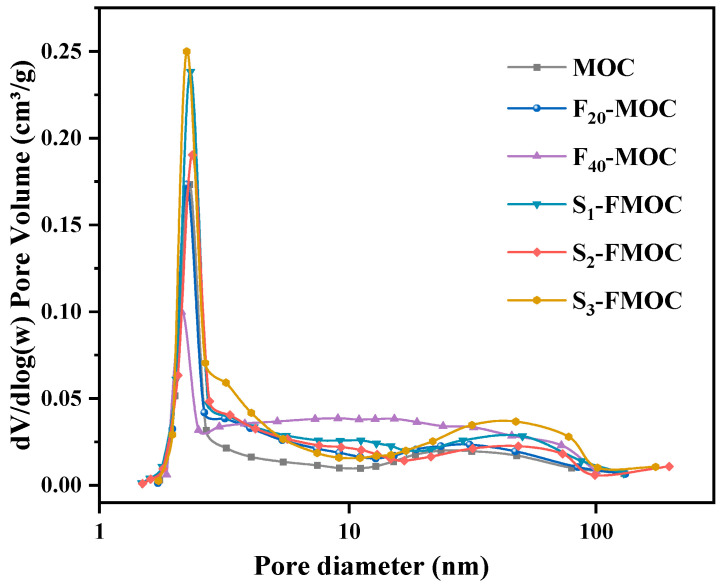
Log differential intrusion curves of hardened multi-doped MOC.

Figure 17 shows the dV/dlogD pore-volume distribution diagram of BJH adsorption of the MOC, F_20_-MOC, F_40_-MOC, S_1_-FMOC, S_2_-FMOC, and S_3_-FMOC samples. It can be seen from the figure that the pore size of the six samples reached the highest value in the region of 3–4 nm. When fly ash and HDTMS were incorporated, the content of pores with 3–4 nm in diameter increased, aiding in the formation of a fine microstructure in MOC. Adding 20% fly ash can fill the pores and separate the large pores into smaller holes, resulting in reduced pore size when compared to the MOC reference sample. After adding HDTMS to FMOC, the total pore volume of MOC increased while the pore diameter continued to decrease. However, the large pores will still be divided, the proportion of gel pores will grow and, ultimately, the water resistance of MOC will be improved, due to the filling impact of fly ash and the presence of Mg–Cl–Si–H gel in the hydration product. However, the mechanical properties of MOC are inevitably adversely affected, due to the growing total porosity.

## 5. Conclusions

MOC was modified with fly ash and HDTMS. The condensation time, fluidity, compressive strength, and water resistance of the modified MOC were studied, and the water-resistance enhancement mechanism of the new MOC was studied through SEM, XRD, FTIR, and TG/DSC analyses. The following conclusions were obtained:

1. Mixing fly ash into MOC obviously increased its setting time and fluidity. The spatial reticulation structure of MOC is the main reason for its poor water resistance. The strength residual coefficient of the control sample was only 0.45, while 20% fly ash improved the water resistance of MOC by increasing the strength residual coefficient to 0.59.
2. The compounding of fly ash and HDTMS in MOC prolonged its setting time while enhancing its flow properties, mainly related to the hydrolysis products of HDTMS and its coupling products with other raw materials. Although the compressive strength of the modified specimens of MOC was reduced after compounding, the strength values still remained around 50–65 MPa. However, the residual coefficient of strength of MOC with fly ash and HDTMS in a water environment could be maintained above 0.8.
3. Microscopic analysis allowed us to determine that compounded fly ash and HDTMS promote the formation of hydration product 5-phase and Mg–Cl–Si–H gel, increasing the density of the internal structure of MOC. Furthermore, HDTMS can delay the reaction between water and 5-phase, such that 5-phase can be better retained. At the same time, HDTMS hydrophobically modifies the fly ash, which is beneficial for the uniform dispersion of modified fly ash, blocking the connected pores and reducing moisture intrusion. According to the pore structure analysis, it was found that the harmful pores were significantly reduced, while the proportion of small-sized pores increased. This refined pore structure also contributes to the enhanced water resistance of MOC.

The research results of this paper are expected to be beneficial in promoting the wider research on and application of MOC.

## Figures and Tables

**Table 1:** Composition of fly ash and magnesium oxide.

Material	MgO	SiO_2_	Al_2_O_3_	CaO	Fe_2_O_3_	Na_2_O	K_2_O	P_2_O_5_
MgO	85.21%	4.87%	2.01%	1.58%	1.32%	-	-	0.21%
Fly ash	0.98%	45.32%	35.12%	3.25%	5.12%	0.35%	1.58%	-

**Table 2:** Composition of MgCl_2_·6H_2_O.

Component	MgCl_2_·6H_2_O	SO_4_^2−^	PO_4_^2−^	Ca^2+^	Fe_2_O_3_
Mass fraction	≥98%	≤0.005%	≤0.001%	≤0.05%	≤0.0005%

**Table 3:** Sample proportions in a MOC mixture (wt%, by mass of MgO).

Mixture	Molar Ratio in All Mixtures		Fly Ash *	HDTMS
	MgO/MgCl_2_	H_2_O/MgCl_2_		
MOC (reference sample)	8	13	0	0
F_10_-MOC	8	13	10	0
F_20_-MOC	8	13	20	0
F_30_-MOC	8	13	30	0
F_40_-MOC	8	13	40	0
S_0.1_-FMOC	8	13	20	0.1
S_0.5_-FMOC	8	13	20	0.5
S_1_-FMOC	8	13	20	1
S_2_-FMOC	8	13	20	2
S_3_-FMOC	8	13	20	3

* Fly ash replaces the corresponding proportion of MgO.

## Data Availability

The data used to support the findings of this study are included within the article.
